# Supplementary material for: Potential for the Production of Carotenoids of Interest in the Polar Diatom Fragilariopsis cylindrus
Source: Mar Drugs. 2022 Jul 29;20(8):491. doi: 10.3390/md20080491 (PMC9409807; doi:10.3390/md20080491)
Supplement: Supplementary file 1 [file marinedrugs-20-00491-s001.zip › Table_S1.pdf]

Table S1: Synthesis of all parameters measured in this study; data are the mean values n=3 ± SE. Spectra of the light treatments are available in Fig. S1. Definitions and unites of all parameters are available in Table 2. PUR, photosynthetic useable radiations; LL : DD, photoperiod Light : Dark.

Spectrum	'White'	'White'	445nm	445nm	445nm	445nm	630nm	'White'	445nm
PUR (µmol photons m^{-2} s^{-1})	5.8	11.7	5.8	11.7	23.4	11.7	11.7	11.7	11.7
Photoperiod (LL : DD)	12 :12	12 :12	12 :12	12 :12	12 :12	24 :00	12 :12	12 :12	12 :12
Temperature (°C)	0	0	0	0	0	0	0	7	7
Fx (mg g DW^{-1})	5.34 ± 1.33	4.30 ± 0.28	7.80 ± 0.50	5.67 ± 0.37	3.82 ± 0.60	6.06 ± 0.48	8.95 ± 1.77	6.39 ± 0.74	6.45 ± 0.68
Ddx+Dtx (mg g DW^{-1})	0.81 ± 0.04	0.76 ± 0.12	0.63 ± 0.13	1.12 ± 0.22	1.03 ± 0.13	0.55 ± 0.06	1.73 ± 0.52	0.45 ± 0.1	0.49 ± 0.03
Fx prod. (µg L^{-1} d^{-1})	4.83 ± 0.27	10.49 ± 1.98	12.85 ± 3.46	21.42 ± 2.56	11.55 ± 2.34	13.52 ± 3.12	1.78 ± 0.57	26.5 ± 3.78	43.80 ± 5.05
Ddx+Dtx productivity	2.32 ± 0.24	4.55 ± 0.36	0.61 ± 0.06	2.94 ± 0.54	3.39 ± 0.36	2.91 ± 0.11	0.79 ± 0.62	2.18 ± 0.38	2.96 ± 0.18
Fx yield(µg Wh^{-1})	2.13 ± 0.41	2.40 ± 0.20	4.08 ± 0.15	3.71 ± 0.44	1.16 ± 0.05	2.18 ± 0.41	0.21 ± 0.07	3.25 ± 0.21	7.53 ± 0.87
Ddx+Dtx yield (µg Wh^{-1})	0.48 ± 0.05	0.49 ± 0.04	0.20 ± 0.02	0.51 ± 0.1	0.30 ± 0.03	0.25 ± 0.01	0.09 ± 0.07	0.23 ± 0.04	0.51 ± 0.03
Growth rate (d^{-1})	0.04 ± 0.007	0.17 ± 0.03	0.11 ± 0.04	0.26 ± 0.04	0.20 ± 0.04	0.14 ± 0.04	0.04 ± 0.03	0.20 ± 0.04	0.37 ± 0.03
Cellular Chl *a* (pg $cell^{-1}$)	0.17 ± 0.02	0.19 ± 0.01	0.21 ± 0.03	0.14 ± 0.02	0.08 ± 0.01	0.17 ± 0.01	0.14 ± 0.01	0.13 ± 0.02	0.11 ± 0.01
Cellular C (pg $cell^{-1}$)	10.44 ± 0.81	9.97 ± 1.37	7.01 ± 1.20	9.52 ± 0.12	6.53 ± 0.68	7.40 ± 0.28	4.37 ± 0.70	7.27 ± 1.10	6.86 ± 0.31
Chl *a* / C (mg g^{-1})	16.13 ± 2.76	19.25 ± 4.10	31.30 ± 4.20	15.39 ± 2.14	13.44 ± 2.08	24.50 ± 1.24	28.27 ± 4.28	18.24 ± 2.25	18.06 ± 1.3
C / N (g g^{-1})	7.45 ± 0.99	5.91 ± 0.32	5.46 ± 0.04	6.16 ± 0.15	6.93 ± 0.45	5.42 ± 0.07	4.32 ± 0.45	7.14 ± 0.20	7.37 ± 0.11
F_V/F_M	0.63 ± 0.01	0.65 ± 0.01	0.63 ± 0.03	0.66 ± 0.02	0.55 ± 0.02	0.61 ± 0.02	0.47 ± 0.03	0.61 ± 0.01	0.64 ± 0.01
α	0.39 ± 0.10	0.45 ± 0.02	0.38 ± 0.03	0.44 ± 0.04	0.39 ± 0.02	0.40 ± 0.01	0.15 ± 0.06	0.45 ± 0.06	0.50 ± 0.04
$rETR_{max}$	18.27 ± 4.36	29.36 ± 2.31	18.5 ± 2.02	32.99 ± 2.04	18.73 ± 0.92	14.16 ± 1.43	5.18 ± 2.39	22.29 ± 0.50	29.07 ± 0.65
E_k	49.13 ± 3.99	67.51 ± 5.40	51.50 ± 3.40	77.56 ± 9.80	46.20 ± 1.86	37.11 ± 4.87	39.13 ± 7.93	51.18 ± 7.96	59.03 ± 4.62
E_{opt}	134.6 ± 23.7	204.3 ± 13.4	122.4 ± 14.1	220.0 ± 30.1	145.6 ± 15.6	161.7 ± 16.8	163.4 ± 42.8	177.3 ± 11.0	192.95 ± 3.7
NPQ_{Eg}	0.22 ± 0.06	0.29 ± 0.05	0.28 ± 0.06	0.38 ± 0.09	0.66 ± 0.03	0.30 ± 0.07	0.76 ± 0.02	0.14 ± 0.06	0.22 ± 0.08
NPQ_{max}	1.51 ± 0.23	1.75 ± 0.17	1.52 ± 0.26	2.01 ± 0.15	2.32 ± 0.04	1.51 ± 0.17	1.58 ± 0.09	1.59 ± 0.07	1.87 ± 0.11
Y_{PSII}	0.36 ± 0.003	0.38 ± 0.02	0.27 ± 0.02	0.40 ± 0.02	0.32 ± 0.008	0.33 ± 0.01	0.13 ± 0.02	0.38 ± 0.05	0.49 ± 0.02
Y_{NPQ}	0.14 ± 0.001	0.15 ± 0.02	0.14 ± 0.002	0.16 ± 0.03	0.17 ± 0.02	0.17 ± 0.02	0.08 ± 0.02	0.10 ± 0.05	0.03 ± 0.01
Y_{NO}	0.49 ± 0.03	0.46 ± 0.05	0.58 ± 0.02	0.44 ± 0.01	0.50 ± 0.03	0.50 ± 0.03	0.79 ± 0.007	0.52 ± 0.01	0.48 ± 0.02
