# Supplementary material for: Potential for the Production of Carotenoids of Interest in the Polar Diatom Fragilariopsis cylindrus
Source: Mar Drugs. 2022 Jul 29;20(8):491. doi: 10.3390/md20080491 (PMC9409807; doi:10.3390/md20080491)
Supplement: Supplementary file 1 [file marinedrugs-20-00491-s001.zip › Table_S2.pdf]

	Temperature			Spectrum				PUR			
	0	7		'White'	660 nm	445 nm		5.8	11.7	23.4	
Growth rate (d^{-1})	b	a	$F_{(1,22)}$=28.90; p>0.001	b	b	a	$F_{(2,22)}$=12.76; p>0.001	NS	NS	NS	NS
F_V/F_M	NS	NS	NS	a	b	a	$F_{(2,22)}$=40.46; p>0.001	a	a	b	$F_{(2,22)}$=13.29; p>0.001
NPQ_{gE}	a	b	$F_{(1,22)}$=58.38; p>0.001	c	a	b	$F_{(2,22)}$=61.23p>0.01	c	b	a	$F_{(2,22)}$=60.43; p>0.001
NPQ_{max}	NS	NS	NS	b	a	a	$F_{(2,22)}$=19.66; p>0.001	c	b	a	$F_{(2,22)}$=59.76; p>0.001
Y_{PSII}	a	b	$F_{(1,22)}$=23.50; p>0.001	a	b	a	$F_{(2,22)}$=21.27; p>0.001	c	NS	NS	NS
Y_{NPQ}	a	b	$F_{(1,22)}$=39.55; p>0.001	ab	b	a	$F_{(2,22)}$=10.75; p>0.001	c	b	a	$F_{(2,22)}$=29.55; p>0.001c
Y_{NO}	NS	NS	NS	b	a	b	$F_{(2,22)}$=94.57; p>0.001	c	a	b	$F_{(2,22)}$=27.74; p>0.001c
Fx content (mg g^{-1} DW)	NS	NS	NS	b	a	b	$F_{(2,22)}$=20.07; p>0.001	a	a	b	$F_{(2,22)}$=21.25; p>0.001
Fx productivity (mg g^{-1} L^{-1})	b	a	$F_{(1,22)}$=124,6; p>0.001	c	b	a	$F_{(2,22)}$=19.01;p>0.001	NS	NS	NS	NS
Fx yield (µg Wh^{-1})	b	a	$F_{(1,22)}$=47.79; p>0.001	b	b	a	$F_{(2,22)}$=16.98;p>0.05	ab	a	b	$F_{(2,22)}$=14.00; p>0.001
Dd+Dt content (mg g^{-1} DW)	a	b	$F_{(1,22)}$=17.96; p>0.001	b	a	b	$F_{(2,22)}$=14.71; p>0.001	NS	NS	NS	NS
Dd+Dt productivity (mg g^{-1} L^{-1})	NS	NS	NS	a	b	a	$F_{(2,22)}$=7.604; p>0.01	b	a	a	$F_{(2,22)}$=13.57; p>0.001
Dd+Dt yield (µg Wh^{-1})	NS	NS	NS	a	b	a	$F_{(2,22)}$=5.369; p>0.05	NS	NS	NS	NS

Table S2: Results of 3-way ANOVA analysis followed by Tukey HSD test to compare the effect of the temperature, light spectrum and intensity on the growth rate, photosynthetic potential, fucoxanthin (Fx), and diadinoxanthin and diatoxanthin (Ddx +Dtx) synthesis in *Fragilariopsis cylindrus*. Letters represent clusters of non-significantly different means for the corresponding parameter, **a** being the highest mean values and other letters following in alphabetic order. NS represent non statically different means for the parameter across the treatments. PUR, photosynthetically usable radiations; F_V/F_M, darck-acclimated photochemical efficiency; NPQ_{gE}, non-photochemical quenching at growing PUR intensity; NPQ_{max} (rel. unit), maximal NPQ induced for the highest intensity of the Rapid Light Curve; Y_{PSII} (rel. unit.), Quantum yield of photochemical energy conversion in PS II; Y_{NPQ} (rel. unit.); Quantum yield of regulated non-photochemical energy loss in PS II; Y_{NO} (rel. unit.), Quantum yield of non-regulated non-photochemical energy loss in PS II; DW, dry weigh; $F_{(df,df)}$, *F*-value; df, Degrees of freedom; p, *p*-value.
