# Supplementary material for: Potential for the Production of Carotenoids of Interest in the Polar Diatom Fragilariopsis cylindrus
Source: Mar Drugs. 2022 Jul 29;20(8):491. doi: 10.3390/md20080491 (PMC9409807; doi:10.3390/md20080491)
Supplement: Supplementary file 1 [file marinedrugs-20-00491-s001.zip › Table_S4.pdf]

Table S4 : Culture f/2 and f media composition used in this study, according to Guillard [1], Guillard ans Rhyther [2].

Component	f/2 medium concentration (M) (Guillard [1])	f medium concentration (M) (Guillard and Rhtyher [2])
$NaNO_3$	8.82×10^{-4}	1.76×10^{-3}
NaH_2PO_4 H_2O	3.62×/-*10^{-5}	7.24×10^{-5}
Na_2SiO_3 $9H^2O$	1.06×10^{-4}	2.12×10^{-4}
$FeCl_3$ $6H_2O$	1.17×10^{-5}	2.34×10^{-5}
Na_2EDTA $2H_2O$	1.17×10^{-5}	2.34×10^{-5}
$MnCl_2$ $4H_2O$	9.10×10^{-7}	1.82×10^{-6}
$ZnSO_4$ $7H_2O$	7.65×10^{-8}	1.53×10^{-7}
$CoCl_2$ $6H_2O$	4.20×10^{-8}	8.40×10^{-8}
$CuSO_4$ $5H_2O$	3.93×10^{-8}	7.86×10^{-8}
Na_2MoO_4 $2H_2O$	2.60×10^{-8}	5.20×10^{-8}
Thiamine HCl	2.96×10^{-7}	5.92×10^{-7}
Biotin	2.05×10^{-9}	4.10×10^{-9}
Cyanocobalamin	3.69×10^{-10}	7.38×10^{-10}

1. Guillard, R. R. L., Culture of Phytoplankton for Feeding Marine Invertebrates. In *Culture of Marine Invertebrate Animals: Proceedings — 1st Conference on Culture of Marine Invertebrate Animals Greenport*, Smith, W. L.; Chanley, M. H., Eds. Springer US: Boston, MA, 1975; pp 29-60.
2. Guillard, R. R. L.; Ryther, J. H., Studies of marine planktonic diatoms. I. *Cyclotella nana Hustedt* and *Detonula confervacea* (Cleve) *Gran. Can. J. Microbiol.* **1962,** 8, (2), 229-239.
