# Supplementary figures and images for: Potential for the Production of Carotenoids of Interest in the Polar Diatom Fragilariopsis cylindrus
Source: Mar Drugs. 2022 Jul 29;20(8):491. doi: 10.3390/md20080491 (PMC9409807; doi:10.3390/md20080491)

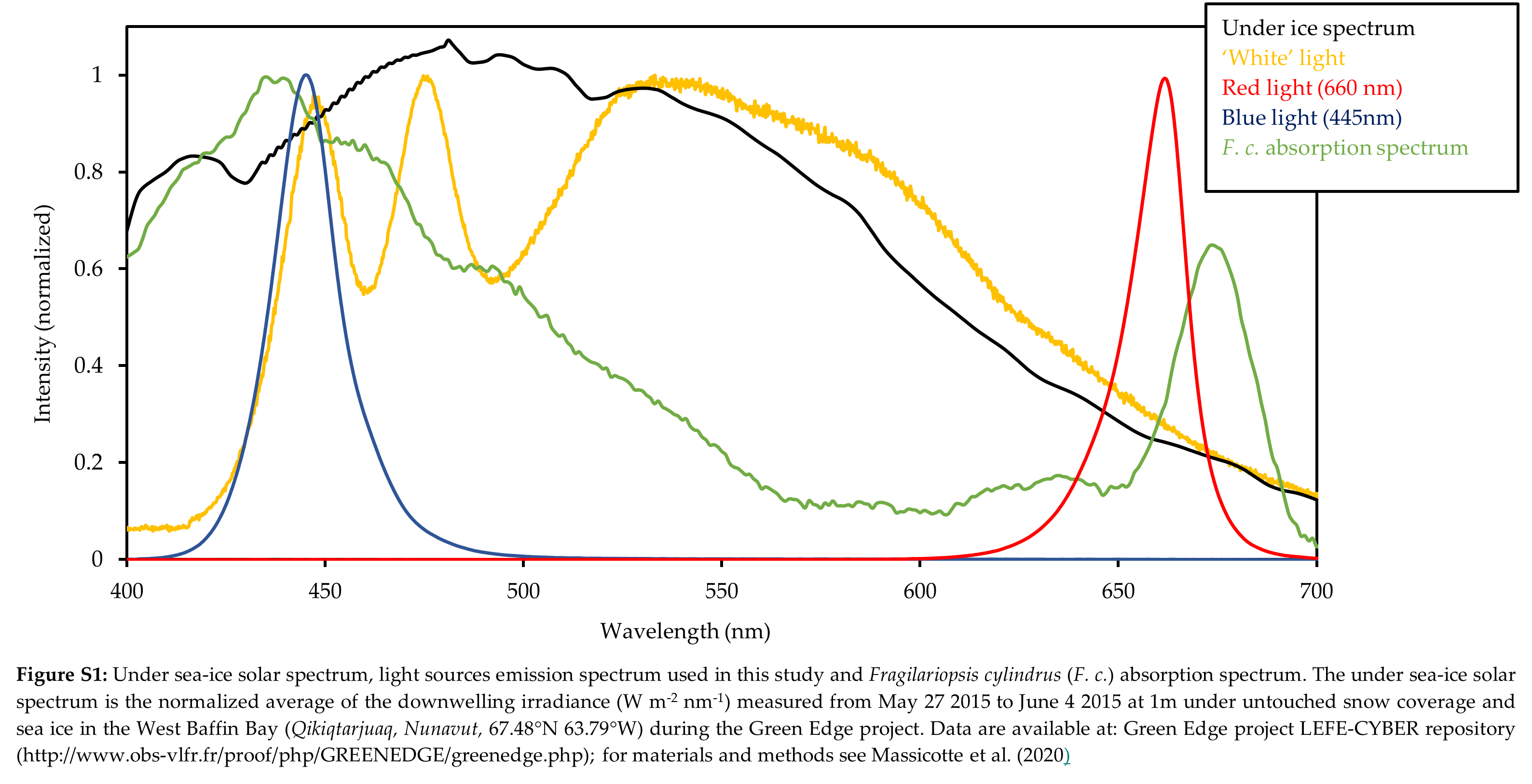

Supplement: Supplementary file 1 [file marinedrugs-20-00491-s001.zip › Figure_S1.png]

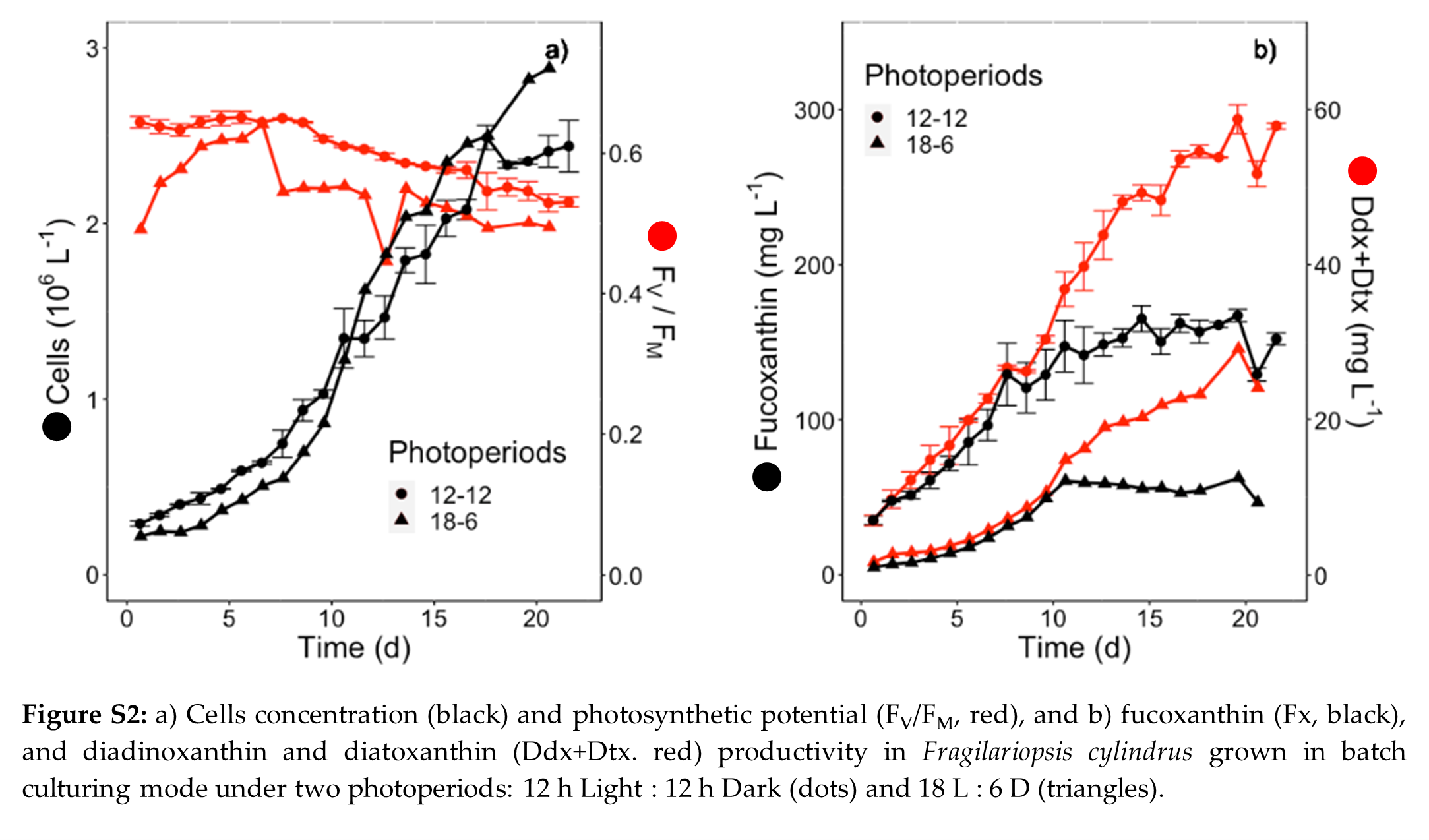

Supplement: Supplementary file 1 [file marinedrugs-20-00491-s001.zip › Figure_S2.png]

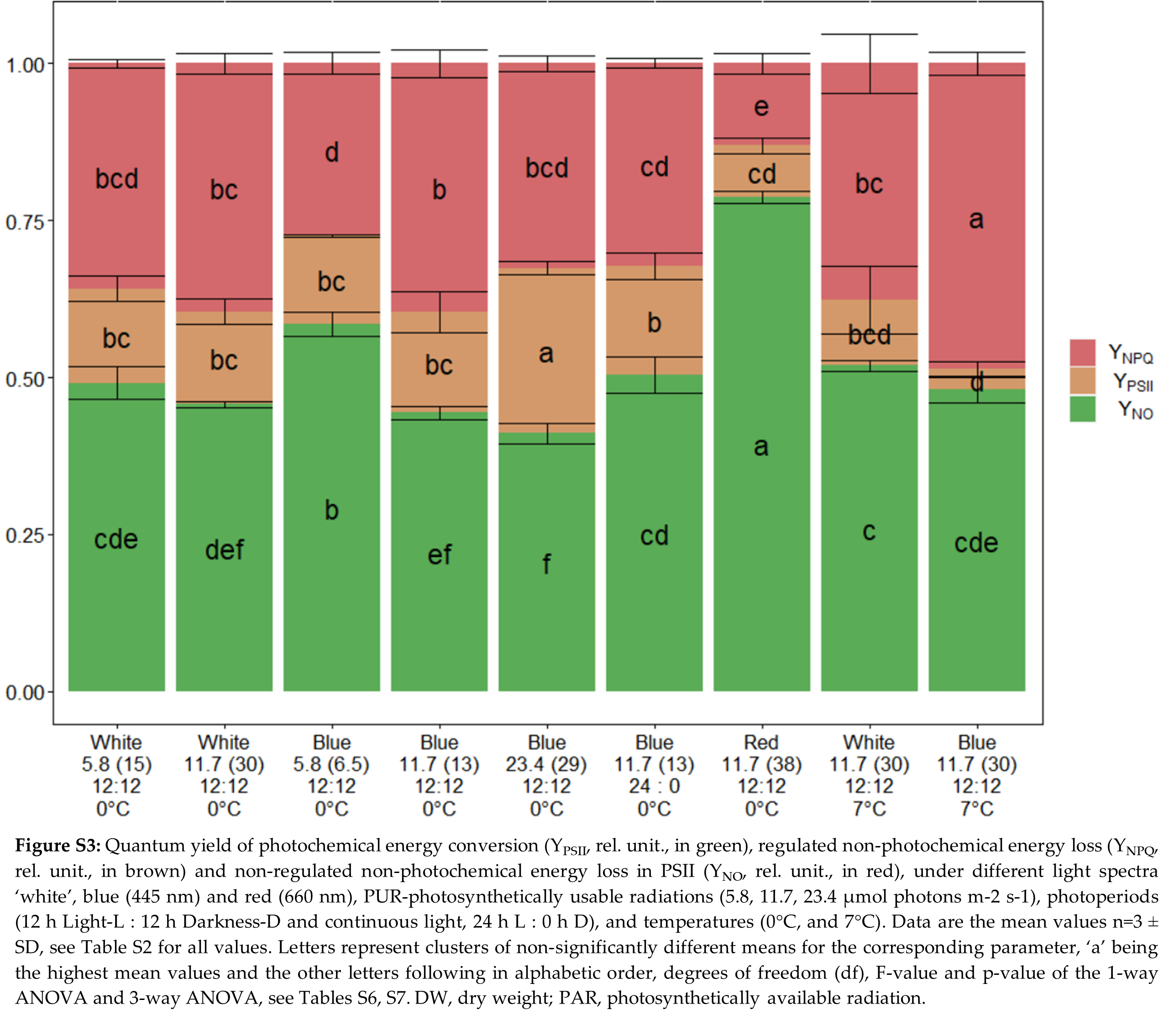

Supplement: Supplementary file 1 [file marinedrugs-20-00491-s001.zip › Figure_S3.png]

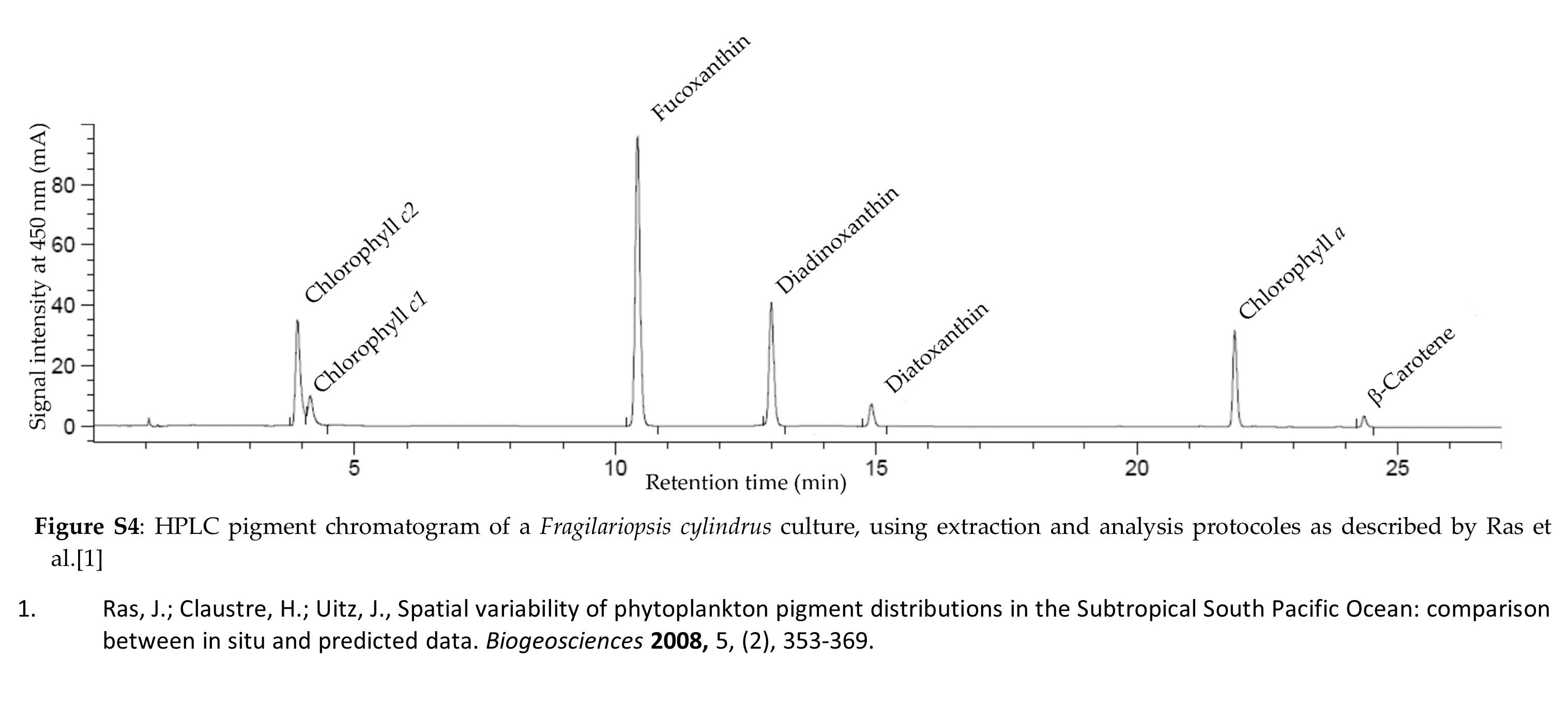

Supplement: Supplementary file 1 [file marinedrugs-20-00491-s001.zip › Figure_S4.png]
